# Stimulation of Human Osteoblast Differentiation in Magneto-Mechanically Actuated Ferromagnetic Fiber Networks

**DOI:** 10.3390/jcm8101522

**Published:** 2019-09-22

**Authors:** Galit Katarivas Levy, Mark A. Birch, Roger A. Brooks, Suresh Neelakantan, Athina E. Markaki

**Affiliations:** 1 Department of Engineering, University of Cambridge, Trumpington Street, Cambridge CB2 1PZ, UK; 2 Division of Trauma and Orthopaedic Surgery, Department of Surgery, University of Cambridge, Addenbrooke’s Hospital, Hills Road, Cambridge CB2 2QQ, UK; mab218@cam.ac.uk (M.A.B.); rb10003@cam.ac.uk (R.A.B.); 3 Department of Materials Science and Engineering, Indian Institute of Technology Delhi, Hauz Khas, New Delhi 110 016, India

**Keywords:** magneto-mechanical actuation, fiber networks, human osteoblasts, mineralization, in vitro osteogenesis

## Abstract

There is currently an interest in “active” implantable biomedical devices that include mechanical stimulation as an integral part of their design. This paper reports the experimental use of a porous scaffold made of interconnected networks of slender ferromagnetic fibers that can be actuated in vivo by an external magnetic field applying strains to in-growing cells. Such scaffolds have been previously characterized in terms of their mechanical and cellular responses. In this study, it is shown that the shape changes induced in the scaffolds can be used to promote osteogenesis in vitro. In particular, immunofluorescence, gene and protein analyses reveal that the actuated networks exhibit higher mineralization and extracellular matrix production, and express higher levels of osteocalcin, alkaline phosphatase, collagen type 1α1, runt-related transcription factor 2 and bone morphogenetic protein 2 than the static controls at the 3-week time point. The results suggest that the cells filling the inter-fiber spaces are able to sense and react to the magneto-mechanically induced strains facilitating osteogenic differentiation and maturation. This work provides evidence in support of using this approach to stimulate bone ingrowth around a device implanted in bone and can pave the way for further applications in bone tissue engineering.

## 1. Introduction

It has long been recognized that the factors governing the changes in bone structure around prosthetic implants are sensitive to microenvironmental cues, consisting of combinatorial biochemical and mechanical stimuli [1,2]. The widely accepted concept of mechanical stimulation-induced bone remodeling [3,4,5] largely forms the basis for rehabilitation and physiotherapeutic exercise regimes applied following implantation of a bone implant. The literature devoted to understanding how mechanical stimuli (fluid flow shear stress, hydrostatic pressure, and deformation by induced stresses/strains) are converted into biochemical signals and then are integrated into cellular responses is vast. Cellular responses have been explained by various mechanotransduction models involving integrin-mediated adhesions and stress activated ion channels [6,7,8]. Table 1 lists the recent studies on cell mechanotransduction as a means of promoting osteogenesis by examining changes to cell signaling and levels of proteins known to be essential for osteogenic differentiation and functional maturation. Although the effects are divergent and difficult to compare because of the different experimental setups/conditions, the studies demonstrate that mechanical loading is a strong regulator of bone biology and function. However, examples of “active” implantable biomedical devices that explicitly include mechanical stimulation as an integral part of their design have been few and far between.

An implant coating/scaffold, made of interconnected networks of slender ferromagnetic fibers sintered together at cross-over points, has been developed to promote the growth of healthy peri-prosthetic bone [9,10]. The design draws on the well-known theories of skeletal physiology and the concepts of strain-regulated bone modelling and remodeling [3,4,5]. According to these theories, the processes of bone remodeling and homeostasis are regulated according to threshold strains, above and below which the respective regulating mechanisms switch on and off. This concept is different from the idea of direct stimulation of bone growth using an externally-applied magnetic field (pulsed electromagnetic fields (see review [11]) or static magnetic fields, e.g., [12,13]). It also differs from the idea of using magnetic fields to generate forces within cells attached to micro- or nano-scale ferromagnetic beads (e.g., [14,15,16,17,18,19]).

**Figure 1:**
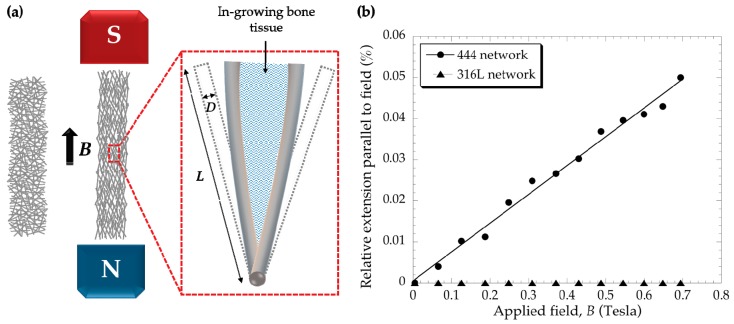
(**a**) Schematic representations of the elastic deformation of a fiber network under a magnetic field *B*. Also shown is the deflection of a bonded pair of fibers deforming in-growing bone tissue. (**b**) Representative relative net extension in the direction of the applied field as a function of the applied magnetic field *B* for 444 and 316L fiber networks (rectangular beam sample clamped at one end).

When the scaffold is actuated by an external magnetic field, it deforms elastically as the ferromagnetic fibers tend to align in the direction of the field, applying strains to in-growing bone tissue (Figure 1a). Because of shape anisotropy, long fibers magnetize easier along their long axis, as the demagnetizing field is negligible in that direction. To estimate the deformations induced in a ferromagnetic fiber network by application of a magnetic field, an analytical magneto-mechanical model has been developed [9,10]. An important (controllable) design parameter is the fiber segment aspect ratio (the sections between the joints *L* over the fiber diameter *D*) as the strain that the fibers can transfer to the in-growing bone is dependent on how much the fibers are able to deflect. For metal fiber networks with relatively low fiber volume fraction and slender fiber segments between joints, the macroscopic magneto-mechanical response of such materials can be predicted using an affine model [9,10] based on the deflection of individual fiber segments subjected to a magnetically-induced bending moment. A prosthesis design allowing such an effect to be exploited would involve a circumferentially proximal porous layer, comprising ferromagnetic fibers, bonded to a conventional non-magnetic stem [20]. Treatment would involve exposure to a magnetic field lower than those used for diagnostic purposes (e.g., MRI) shortly after the implant operation, which is the critical period for bone ingrowth.

Ferritic stainless steels are strong candidates for this application because they show relatively high magnetic inductions. One potential ferritic stainless steel grade is 444; a steel with very low additions of carbon and nitrogen, and a small amount of strong carbide elements such as Nb and Ti to improve corrosion resistance [21,22]. 444 was found [23] to elicit comparable in vitro cellular responses in terms of osteogenesis, toxicity, and inflammatory reaction to 316L in short-term culture.

This study addresses the hypothesis that cell response can be influenced by magneto-mechanical strain induction via a ferromagnetic fiber network. Here, we show for the first time that this type of actuation increases matrix production, mineralization, and osteogenic gene and protein expression in human osteoblasts cultured onto ferromagnetic fiber networks.

## 2. Experimental Section

### 2.1. Substrates—Fibre Networks

This study utilized two types of solid-state sintered stainless-steel fiber networks made of 444 ferritic stainless steel (Nikko Techno Ltd., Tokyo, Japan) and 316L austenitic stainless steel (Bekaert SA, Zwevegem, Belgium), containing ~15 vol% of fibers (Table 2). 444 networks were produced by shaving 60 µm fibers off a 100 µm thick coiled metal foil, which led to a rectangular cross-sectional shape. The 316L networks were made of bundle-drawn fibers in a hexagonal cross-sectional shape. The diagonal length of the fiber cross-sectional area was about 40 µm. Details on the manufacturing of these networks are described elsewhere [40,41]. Information on the 444 and 316L networks are presented in Table 2 [41,42]. Although the fiber volume fractions and fiber orientation distributions are very similar between the two networks, as illustrated in Table 2, there are differences in the fiber cross-sectional shape and size. Because the 444 networks are comprised of coarser fibers (60 × 100 µm^2^), they have less fibers per unit area, and consequently larger inter-fiber spaces compared to the 316L networks, of the same fiber volume fraction, made of finer fibers (diagonal length 40 µm, side length 20 µm). Because of these differences, 316L networks were included in the study as a control, rather than an experimental group.

**Figure 2:**
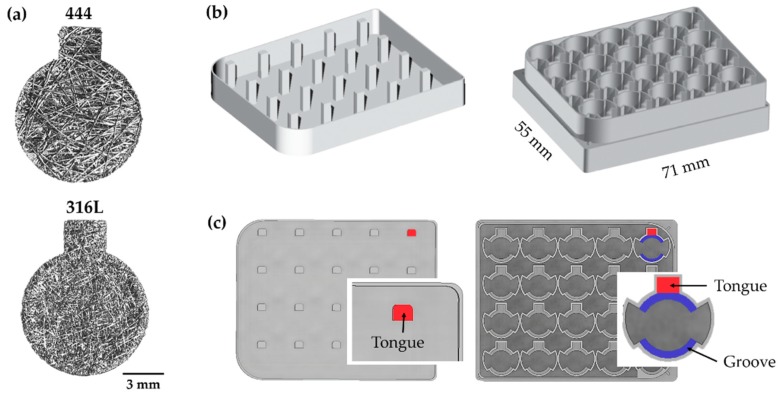
(**a**) 444 and 316L fiber networks, cut in a keyhole shape, for magnetic actuation. (**b**,**c**) Views of the lid and base of the bespoke culture plates. The lid contains tongues (red) to grip the samples, the latter are resting on grooves (blue) at the well mid-height.

For all experiments, ~1 mm thick sheets were used. For the cell-seeded magnetic actuation experiments, samples were cut into a keyhole shape (Figure 2a) using a bespoke die press. The samples had a diameter of 9 mm and a rectangular gripping section (2.3 × 3.0 mm^2^). They were ultrasonically cleaned for 15 min sequentially in acetone, ethanol, and ultrapure water, dried in air at room temperature followed by sterilization at 126 °C for 20 min using a Prestige Medical™ Classic Autoclave. For the magnetic deflection experiments, rectangular beam samples with dimensions of 40 mm (L) × 10 mm (w) × 1 mm (t) were cut by electro-discharge machining.

### 2.2. Cell Culture and Seeding

Fetal human osteoblasts (fHOb), obtained from the European Collection of Cell Cultures (406-05f, ECACC), were selected for the cell culture studies. Cells were maintained in McCoy’s 5A medium (Gibco™, 16600082), supplemented with heat-inactivated 10% FBS (Invitrogen, 10108-157) and 1% antibiotic-antimycotic (Gibco™, 15240062) and 50 mg·mL^−1^ L-ascorbic acid phosphate magnesium salt (FUJIFILM Wako Chemical corporation, 013-19641). Trypsin-EDTA solution (Sigma, T4049) was used to detach the cells for cell passaging and seeding. fHObs in the third passage were used for all experiments. Prior to seeding, the sterilized networks were pre-wet by immersing in culture medium. The networks were then placed onto sterile hydrophobic PTFE (polytetra-fluoroethylene) membranes with 5 μm pore size (Fisher Scientific, 10676741). A 75 μL droplet of cell medium containing 7.5 × 10^4^ cells was placed on top of each network and the networks were then incubated for 4 h at 37 °C in a humidified atmosphere of 5% CO_2_ to allow cell attachment. After this period, the networks were each transferred to a well of a 24–well plate, covered with 1 mL of culture medium. To induce differentiation, 10 nM dexamethasone (Sigma, D2915) and 10 mM β-glycerophosphate (Fisherscientific, 10424701) were added to the culture medium after 2 days in accordance with previous work [43]. The medium was replenished every other day.

### 2.3. Cell Adhesion and Cytoskeleton Organization

The samples were fixed with 4% (*w*/*v*) paraformaldehyde (Sigma–Aldrich, Haverhill, UK, 252549) for 15 min at room temperature, permeabilized with 0.1% Triton X-100 (Sigma, T878) and 0.1% TWEEN-20 (Sigma–Aldrich, Haverhill, UK, P6585) for 15 min and incubated with 1% (*w*/*v*) bovine serum albumin (BSA) in phosphate-buffered saline (PBS) at 37 °C for 10 min to block non-specific binding. The actin cytoskeleton was visualized using FITC-labelled phalloidin (1:100) (Sigma, P5282) followed by cellular nuclei staining with Vectashield antifade mountant containing 4,6-diamidino-2-phenylindole (DAPI), (Sigma, F6057). The fluorescence images were obtained using a Zeiss Axio-Obsorber.Z1 fluorescence microscope.

### 2.4. Cell Mineralization

#### 2.4.1. OsteoImage^TM^ Mineralization Assay

Mineralization was assessed using the OsteoImage^TM^ Mineralization Assay (Lonza, PA-1503) according to the manufacturer’s instructions. This assay is based on the specific binding of a fluorescent staining reagent to bone-like minerals deposited by cells. Briefly, cells were fixed with 4% formaldehyde in PBS for 30 min at room temperature and stained with the staining reagent (1:100 in staining reagent dilution buffer) for 30 min, protected from light. Samples were washed with the washing buffer and mounted with DAPI. The hydroxyapatite of the bone nodules was measured qualitatively using a Zeiss Axio-Obsorber.Z1 fluorescence microscope and quantitatively by ImageJ software (7 images for a sample, 3 samples per group, National Institutes of Health, Maryland, USA).

#### 2.4.2. Alizarin Red Staining

Alizarin red staining (Sigma–Aldrich, Haverhill, UK, A5533) was used to visualize the calcium deposition indicating mineralization of cells after 16 and 21 days for all magnetic and static groups. Briefly, the samples were washed with PBS and fixed in 4% (*v*/*v*) formaldehyde at room temperature for 30 min. After washing with excess distilled water (dH_2_O), Alizarin red solution (2% *w*/*v* in dH_2_O adjusted to pH 4.2 using 0.5% ammonium hydroxide) was used to cover the samples for 30 min. After aspiration of the unincorporated dye, the samples were washed thoroughly with dH_2_O. The samples were visualized using a Zeiss Axio-Obsorber.Z1 fluorescence microscope (fluorescence emission 580 nm). To assess relative levels of matrix mineralization the Alizarin Red stain was extracted from the samples (3 samples per group) by adding 800 µL of 10% acetic acid followed by 30 min incubation at room temperature as described previously [44]. The absorbance at 405 nm of the solubilized Alizarin red dye from the samples was measured using a FLUOstar^®^ Omega plate reader (BMG Labtech Ltd., Aylesbury, UK). An Alizarin red staining standard curve was established with a known concentration of the dye.

### 2.5. Gene Expression Analysis

On day 21 of culture, the total RNA was extracted from the cell-seeded scaffolds using RNeasy Protect Mini Kit (Qiagen, Manchester, UK, 74124) according to the manufacturer’s instructions. Complementary deoxyribonucleic acid (cDNA) was synthesized by reverse transcriptase–polymerase chain reaction (RT-PCR) using QuantiTect^®^ Reverse Transcription Kits (Qiagen, 205311) according to the manufacturer’s instructions. RT-PCR was conducted using the QuantiFast SYBR Green PCR Kit (Qiagen, 204056) with the following primers: glyceraldehyde-3-phosphate dehydrogenase (*GAPDH*), bone morphogenetic protein 2 (*BMP-2*), collagen type 1α1 (*COL1A1*), alkaline phosphatase (*ALP*), osteocalcin (*OCN*), runt-related transcription factor 2 (*Runx2*), and vascular endothelial growth factor (*VEGF*), which amplify transcripts characteristic of osteoblasts. The primers were obtained from Qiagen and reconstituted according to the manufacturer’s instructions. The cycle conditions were performed with a 5 min at 95 °C activation step followed by 40 cycles with 10 s at 95 °C denaturation and 30 min at 60 °C extension step. *GAPDH* expression served as an internal control. Relative expression was calculated using the 2^−ΔΔCT^ method according to Livak and Schmittgen [45]. Results were presented as fold change expression normalized to their non-actuated counterparts to determine the effect of the magneto-mechanical actuation.

### 2.6. Protein Release

Human *BMP-2* (Sigma–Aldrich, Haverhill, UK, RAB0028-1KT) and human osteocalcin (Sigma–Aldrich, Haverhill, UK, RAB1073-1KT) were used according to the manufacturer’s instructions for quantitative measurement of the chosen proteins in the cell culture supernatants. Briefly, cell culture media were removed after 21 days, pipetted in polypropylene Eppendorf tubes and stored at −80 °C until analysis in an enzyme-linked immunosorbent assay (ELISA). The antibodies, employed in this assay, were specific for human *BMP-2* and *OCN* and were coated on the 96-well plate provided. Standards and samples from the fiber networks were pipetted into the wells and *BMP–2/OCN* present in a sample was bound to the wells by the immobilized antibody. The wells were washed and biotinylated anti-human *BMP–2* or *OCN* antibody were added. After washing away the unbound biotinylated antibody, HRP-conjugated streptavidin was pipetted to the wells. The wells were washed again, a TMB substrate solution was added to the wells and color developed in proportion to the amount of *BMP–2* and *OCN* bound. The stop solution changed the color from blue to yellow, and the intensity of the color was measured as absorbance using the FLUOstar^®^ Omega plate reader (BMG Labtech Ltd., Aylesbury, UK) at 450 nm. With the help of a standard curve, the protein content was determined and set in relation to the respective controls.

### 2.7. Magneto-Mechanical Actuation

#### 2.7.1. Magneto-Mechanical Actuation of Cell-Seeded Fiber Networks

The cell actuation experiments were carried out using a 1.6 Tesla DC coil electromagnet, with a homogeneous operating region of 75 × 75 × 75 mm^3^, designed by Hirst Magnetic Instruments Ltd. (Cornwall, UK). The field coils were powered by an 11 KW 30 V/500A power supply with an internal main breaker of three modules of 40 A each. A customized OKO-lab incubating chamber was supplied by Indigo Scientific Ltd. (Herts, Baldock, UK). The system was an electrically-heated CO_2_ incubator equipped with temperature, gas, and humidity controllers. CO_2_ levels inside the chamber were maintained at 5% by purging with a 5% CO_2_/air medical gas mixture.

Cell culture plates: Bespoke polystyrene cell culture plates were produced by micro-machining (Protolabs, Telford, UK). A schematic depiction of the plates is shown in Figure 2b,c. Figure 2b shows the lid and base of the bespoke culture plates. The lid contained tongues (marked in red, Figure 2c) which were used to grip the rectangular section of the keyhole samples while the circular area of the keyhole samples (Figure 2a) was free to deform during the actuation process. The samples were resting on grooves (Figure 2c—blue) at mid-height of the well providing intra-well space for medium flow and ease in sample handling. An offset spacer was introduced in the plate corner, to allow a 0.5 mm gap for air circulation when the base of the plate was covered with the lid. Air circulation allowed equilibration of the well medium with the gas phase (humidified atmosphere of 5% CO_2_/95% air). The temperature was maintained at 37 °C during the actuation process. This was verified by measuring in situ the media temperature of the wells containing the actuated fiber networks and also the air temperature above the polystyrene cell culture plates.

Actuation protocol: All actuation experiments involved static culture for 7 days (to allow cells to colonize the inter-fiber spaces), followed by daily actuation for 5 hr (equivalent to 3600 cycles per day) for 14 days. The magnetic field varied sinusoidally from 0.3 to 1.1 Tesla at a frequency of 0.2 Hz. In all studies, the response of 444 magneto-mechanically actuated networks (444_M) was normalized against the control samples (444 non-actuated networks, 444_S). To investigate any direct effects of the magnetic field on the cells, 316L (non-magnetic) networks underwent the same actuation protocols (316L_M) and were compared to non-actuated 316L samples (316L_S).

#### 2.7.2. Magneto-Mechanical Network Deflection

Rectangular beam samples were magneto-mechanically actuated using a 0.7 Tesla coil electromagnet. The setup involved two water-cooled coils, which were separately powered using a 60V-20A DC power supply. The samples were placed between the pole pieces of the magnet, with their long axis parallel to the applied magnetic field, and held in a mechanical clamping arrangement, secured at one end (Supplementary section, Figure S4). The field strength was ramped up stepwise by increasing the voltage of the power supply and the applied magnetic field strength was measured using a Hirst transverse hall probe. The corresponding deflections along the length (i.e., parallel to the applied field) were captured using a laser scanning extensometer (Model: Beta Lasermike, AS1000; resolution of ±1 μm). Four samples were tested. Note: The magnet employed for magneto-mechanical actuation of cell-seeded networks could not be used for the deflection experiments, as it did not allow the receiver and transmitter of the extensometer to be located away from the magnetic pole pieces thereby affecting the laser extensometer readings. As a result, network deflections could only be measured for up to 0.7 Tesla (instead of 1.1 Tesla), which was the maximum field this magnet could achieve.

### 2.8. Statistical Analysis

The results are expressed as mean ± standard error from three independent experiments (*n* = 3). Differences between the actuated and non-actuated groups were determined by unpaired *t* test using Prism 8^®^, Graph-Pad Software Inc (San Diego, CA, USA). The threshold for statistical significance was set at a value of *p* < 0.05.

## 3. Results

### 3.1. Fibre Network Deformation

Figure 1b shows the relative length changes for 444 and 316L fiber networks as the applied magnetic field was increased. The applied field was ramped up to a maximum of ~0.7 Tesla. The relative length extension for the 444 networks reached up to ~0.05%, for the maximum applied field. As expected, 316L (non-magnetic) networks show no extension.

### 3.2. Cell Adhesion and Cytoskeleton Organization

**Figure 3:**
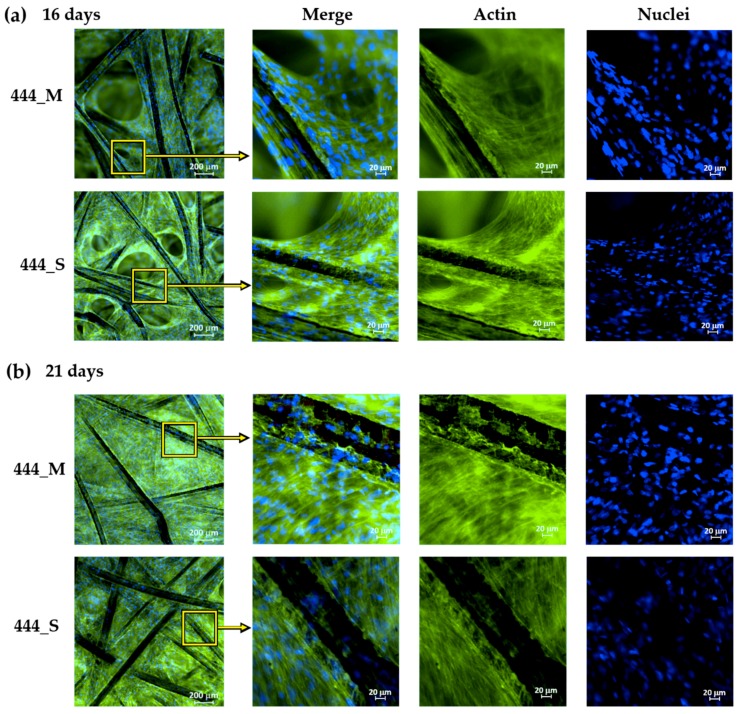
Representative immunofluorescence images showing magnetically-actuated (M) and static (S) 444 fiber networks on day (**a**) 16 and (**b**) 21 of culture. FITC-phalloidin and DAPI were used to stain the actin cytoskeleton green and the nuclei blue, respectively.

Figure 3 shows the immunofluorescence staining of the actin filaments in the cytoskeleton and cell nuclei for the 444 networks. On day 16 of culture, the cells started to populate the inter-fiber spaces and form bridges between adjacent fibers (Figure 3a). On day 21, the cells have filled the inter-fiber spaces of the networks (Figure 3b). This was observed throughout the thickness of the fiber networks, as illustrated in the cross-sectional images (Supplementary section, Figure S1). The cells showed a well-organized actin cytoskeleton. A similar response was observed in static and actuated 316L networks (Supplementary section, Figure S2).

### 3.3. Cell Mineralization

**Figure 4:**
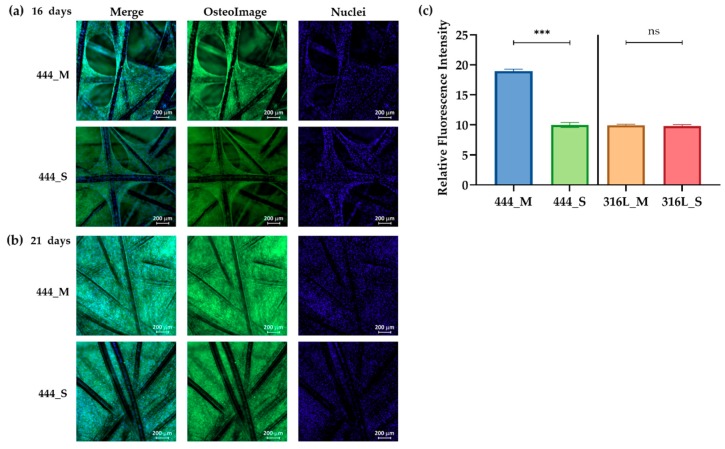
Fluorescence imaging of mineralization obtained using the OsteoImage Mineralization Assay, for 444 actuated and non-actuated networks on days (**a**) 16 and (**b**) 21 of culture. OsteoImage is a fluorescent assay that stains bone-like minerals (including hydroxyapatite) in green and DAPI (blue) was used to counter-stain the fixed cells. (**c**) Quantification of relative staining intensities on day 21 day for all the tested groups using ImageJ. Bars represent the mean ± standard error for each tested group (*n* = 3 independent experiments). Statistical analysis was conducted by unpaired *t* test, *** *p* < 0.001, ns: no statistical significance.

Mineralization was visualized and quantified using the fluorescence-based OsteoImage Mineralization Assay. Figure 4a shows the images of stained 444 networks on days 16 and 21 of culture. The presence of bone mineral (green fluorescence staining) is apparent in both static and actuated networks after prolonged time in culture. The amount of mineralization occurring in both networks was quantified on day 21, and it is clear from the data in Figure 4b that the 444 actuated networks had a significantly higher bone mineral content compared to non-actuated networks, while no statistically significant differences were observed between the 316L networks (actuated and non-actuation, *p* = 0.7).

**Figure 5:**
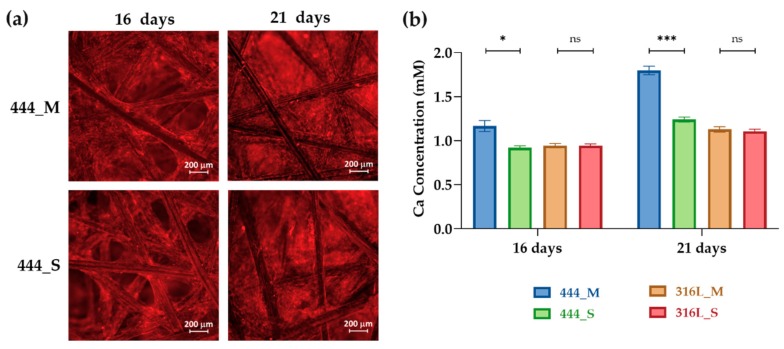
(**a**) Fluorescence imaging of calcium-rich deposits, stained with Alizarin red, of 444 actuated and non-actuated networks on days 16 and 21 of culture. Red areas indicate positive staining for calcium-rich deposits. (**b**) Calcium concentration on days 16 and 21 for all the tested groups measured from the released Alizarin red stain using a plate reader at 405 nm. Bars represent the mean ± standard error for each tested group (*n* = 3). Statistical analysis was conducted by unpaired *t* test, * *p* < 0.05, *** *p* < 0.001, ns: no statistical significance.

To further analyze the production of the extracellular matrix by the osteoblasts, Alizarin red staining was used to relatively quantify the amount of calcium deposition as presented in Figure 5. It can be seen that the osteoblasts undergo osteogenic differentiation by producing mineralized nodules in both 444 actuated and non-actuated groups on days 16 and 21 (Figure 5a). Staining revealed a thick and dense calcium-rich (red) layer of new mineral matrix synthesized by the osteoblasts. Figure 5b shows higher calcium concentrations in the 444 actuated networks compared to the non-actuated (control) group on days 16 and 21 of culture. Again, no statistically significant differences were observed between the actuated and non-actuated 316L networks (*p*_(16 days)_ = 0.52, *p*_(21 days)_ = 0.37).

### 3.4. Gene Expression Analysis

**Figure 6:**
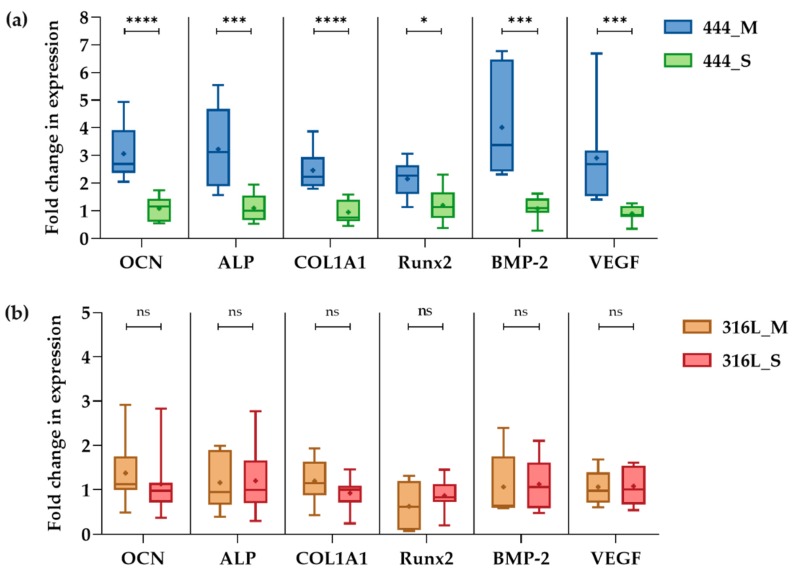
RT-PCR analysis of osteogenic gene expression of human osteoblasts after 21 days of culture for (**a**) 444 and (**b**) 316L fiber networks. The networks were cultured statically for 7 days followed by a daily actuation for 14 days using a magnetic field varying from 0.3 to 1.1 Tesla at a frequency of 0.2 Hz for 5 h. Boxes show interquartile ranges, horizontal lines within the boxes indicate the median, + shows the mean and whiskers denote minimum and maximum values (*n* = 3, 3 samples for each group per experiment). Data are reported in an x-fold expression of static cultures. Statistical analysis was conducted by unpaired *t* test, * *p* < 0.05, *** *p* < 0.001, **** *p* < 0.0001, ns: no statistical significance. Osteocalcin, *OCN*; alkaline phosphatase, *ALP*; collagen type 1α1, *COL1A1*; runt-related transcription factor 2, *Runx2*; bone morphogenetic protein 2, *BMP-2*; vascular endothelial growth factor, *VEGF*.

The effect of magneto-mechanical actuation on the differential expression of osteogenic genes was quantified using RT-PCR analysis. As illustrated in Figure 6a, on day 21 of culture, actuated 444 networks show an upregulation of *OCN* (3.1–fold clustered mean across the three independent studies), *ALP* (3.2–fold), *COL1A1* (2.5–fold), *Runx2* (2.2–fold), *BMP-2* (4–fold), and *VEGF* (2.9–fold) compared to non-actuated networks. There were no statistically significant differences in gene expression between 316L actuated and non-actuated groups across the three independent studies (Figure 6b).

### 3.5. Protein Release

**Figure 7:**
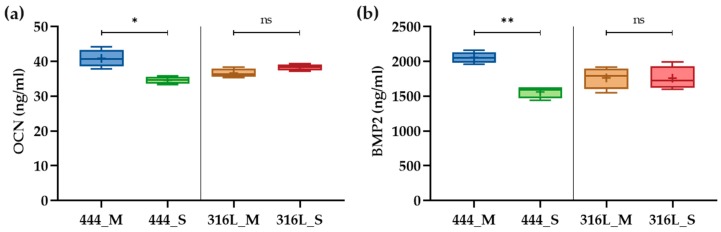
ELISA quantification of bone protein concentration in cell culture supernatant by human osteoblasts on day 21 of culture: (**a**) *OCN* and (**b**) BMP-2. Boxes show interquartile ranges, horizontal lines within the boxes indicate the median, + shows the mean and whiskers denote minimum and maximum values (*n* = 3, 2 samples for each group per experiment). Statistical analysis was conducted by unpaired *t* test, * *p* < 0.05, ** *p* < 0.01, ns: no statistical significance.

*OCN* and *BMP-2* protein secretion by the osteoblasts was measured using ELISA. On day 21 of culture, 444 actuated networks showed a 1.2- and 1.3-fold increase in supernatant *OCN* and *BMP-2* concentrations respectively compared to the 444 non-actuated networks (Figure 7). There were no statistically significant differences in *OCN* and *BMP-2* release between the actuated and non-actuated 316L networks.

## 4. Discussion

The purpose of this study is to determine whether a magneto-active layer, which could be incorporated in the proximal region of the implant, can mediate osteogenesis in vitro. The layer is composed of an interconnected network of slender ferromagnetic fibers, which can be actuated in vivo by the application of an external magnetic field (of clinical magnitude) causing the network to deform elastically applying strains to in-growing cells. Deformation of this type has been analyzed previously [10] using an analytical model based on the deflection of a single ferromagnetic fiber in a magnetic field. Figure 1b shows the relative length change of the networks as a function of the applied magnetic field with a maximum value of 0.7 Tesla. The rate of strain increase varies linearly with the applied field, which is in agreement with the predicted shape changes (Supplementary section). It also shows that if the strains follow the trend shown in Figure 1b, length changes of around 0.1% could be generated using this particular fiber network for a magnetic field of 1.1 Tesla. However, significantly higher strains can be generated by using networks with higher fiber (segment) aspect ratios (distance between joints *L*/fiber diameter *D*) and also by increasing the magnitude of the imposed magnetic field *B*.

The actuation strategy employed in this work involved static culture of fetal human osteoblasts seeded onto 444 ferritic stainless steel networks for a week, to allow human osteoblasts to populate the network inter-fiber spaces, followed by a two-week daily actuation using an external magnetic field varying sinusoidally between 0.3–1.1 Tesla at 0.2 Hz for a period of 5 h. 316L networks of the same porosity, but with different fiber geometry and size (Section 2.1), were also used in the study. Because of these differences, they were included as a control group, rather than an experimental group, in order to investigate any direct effects of the magnetic field on the cells. On day 2 of culture, the networks were cultured in the presence of osteogenic supplements (dexamethasone and β-glycerophosphate).

Immunofluorescence staining on days 16 and 21 (Figure 3) displayed marked cell growth in the inter-fiber spaces as the cells were able to migrate or stretch further away from the fibers and spread across the inter-fiber spaces in both static and actuated 444 networks. On day 21, the cells populated the inter-fiber spaces throughout the fiber networks.

The mineralization process and the production of the extracellular matrix (ECM) in osteogenic cell culture can be detected by the formation of mineralized nodules composed of inorganic hydroxyapatite and organic components including type I collagen [46]. In this study, two complementary assays were carried out. OsteoImage^TM^ mineralization assay (Figure 4) which binds to the mineral component of the bone-like nodules deposited by cells, and Alizarin red staining that detects calcium accumulation and formation of chelates (Figure 5) [47]. Both assays showed that mechanical actuation stimulated mineralization and ECM production as demonstrated by the increased amount of bone-like mineral compared to static 444 networks (*p* < 0.001). Staining is punctate as observed previously [23]. No differences were observed between actuated 316L networks when compared to 316L networks cultured under static conditions (*p* > 0.3). These findings were supported by upregulation of the *COL1A1* by 2.5-fold (*p* < 0.0001), shown in Figure 6a, for the 444 actuated group compared to the 444 static group. *COL1A1* is the main component in osseous ECM [48]. It can mediate cell adhesion, contribute to the mature osteoblast phenotype, and provide a template for mineralization [49].

To further analyze the effect of magneto-mechanical actuation on the osteoblast osteogenic capacity, bone formation markers such as *ALP* and *OCN* as well as *Runx2*, *BMP-2*, and *VEGF*, which have known functions in bone biology, were evaluated (Figure 6 and Figure 7). *OCN* is the second most abundant protein in bone after collagen that can be found in a fully-mineralized matrix (late markers of osteoblast differentiation) and promotes deposition of mineral substance [50]. *ALP* is an ectoenzyme, highly expressed in active osteoblasts [46] and plays a role in bone mineralization by controlling the concentrations of mineralization inhibitors and phosphate ions. In this study, both *ALP* (3.2-fold (*p* < 0.001)) and *OCN* (gene-level: 3-fold (*p* < 0.0001), protein-level: 1.2-fold (*p* < 0.05)) were upregulated for the 444 actuated group compared to the static group across the three independent studies, while no significant differences were observed in the 316L groups. These results are in line with previous studies [51,52,53], that reported upregulation of the above markers at both gene and protein levels upon application of mechanical stimulation and have previously been associated with calcium binding and bone matrix mineralization process.

*Runx2* is an essential transcription factor for osteoblastic differentiation and bone formation, and directly regulates the expression of *OCN* [54]. Also, *Runx2* mutations in humans, that reduce the level of this gene’s functional activity, are responsible for cleidocranial dysplasia [55]. In this study, mechanical actuation increased the *Runx2* expression by 2.2-fold (*p* < 0.05). This is in line with previous reports [34,56,57], that demonstrated upregulation of *Runx2* as a result of mechanical stimulation such as cyclic compression (5 and 10% strain) and tension (1%). *BMP-2* is accumulated in ECM and is shown to stimulate osteoblastic differentiation in vitro [58]. It exhibits this osteogenic action by regulating transcription of osteogenic genes such as *ALP*, *COL1A1*, and *OCN*. Moreover, *BMP-2* is known to control the expression and functions of *Runx2* [54]. For example, osteoblasts cultured onto polycaprolactone (PCL) fiber networks showed an upregulation of *BMP-2* (17-fold), *Runx2* (1.6-fold), *ALP* (2.5-fold), *COL1A1* (2.2-fold), and *OCN* (3.4-fold) compared to static networks when the scaffolds, after 4 weeks of culture, were subjected to 10% cyclic compressive strain at 0.5 Hz for 4 h [57].

*VEGF* is the main angiogenic growth factor involved in bone healing and has an important role in bone repair by stimulating osteoblasts [46,59]. A synergy between *BMP-2* and *VEGF* has been reported [60], in which there is an intimate relation to bone development and healing that is advantageous for bone regeneration procedures. In the present study, *BMP-2* was upregulated by four–fold while magnetic actuation augmented the expression of *VEGF* by 2.9-fold (*p* < 0.001). Also, a 1.3–fold increase in supernatant *BMP-2* concentration was measured compared to the static networks (*p* < 0.01).

Magneto-mechanical actuation of 316L (non-magnetic) networks showed a similar deposition of bone-like minerals, gene expression, and protein release to the supernatant compared to those cultured without actuation suggesting that the magnetic field has no direct effect on the cellular responses. The effects observed in cells within the 444 (magnetic) networks can be attributed to the mechanical deformations induced in in-growing cells via magneto-mechanical actuation of the fiber networks. During the first weeks of culture, cells form bridges between connecting fibers and will then begin to form cell-matrix accumulations in the corners of the inter-fiber spaces where two fibers meet by curvature driven tissue growth as previously described for MC3T3-E1 mouse osteoblasts [61]. The cells are linked to each other or to the ECM and thereby linked either directly or indirectly to the fibers. In response to the application of a magnetic field, the networks deform elastically, transmitting stresses and strains to in-growing cells via small local fiber deflections. Deformation of the fiber networks will also cause fluid flow within the networks producing shear stresses (tangential frictional forces produced when fluid flows over the cell surface). To induce the changes in cell behavior and matrix deposition, these forces must be converted into biochemical responses by acting through mechanosensitive molecules within focal adhesion complexes, ion channels, and the cytoskeleton [62].

In summary, the results of this study show that mechanical actuation increases the expression of genes important for osteoblast differentiation (*Runx2*, *BMP-2*), matrix deposition, and mineralization (*COL1A1*, *ALP*, *OCN*) and vascularization (*VEGF*). There was a concomitant increase in the amount of *BMP-2* and osteocalcin produced by the cells and enhanced deposition of mineralized matrix. These results demonstrate that the strains produced during magneto-mechanical actuation of ferromagnetic fiber networks can increase the in vitro osteogenic differentiation of human osteoblasts growing within the networks. Future work will focus on the effect of this actuation on self-assembly of endothelial cells and supporting cell populations (e.g., osteoblasts) into vessel-like structures, which is important for bone formation.

## 5. Conclusions

In this study, magneto-mechanical actuation was investigated as a means of promoting osteogenesis in vitro in highly porous ferromagnetic fiber networks by examining mineralization, ECM production, and the resulting expression of genes, proteins, and transcription factors known to be essential for osteogenic differentiation and functional maturation. We showed that the actuation can enhance the mineralization, ECM production, and upregulate osteogenesis by inducing *OCN*, *ALP*, *COL1A1*, *Runx2*, and *BMP-2* expression, and result in the synthesis of proteins involved in osteoblast differentiation and mineralized matrix deposition. Furthermore, actuation increased gene expression of the pro-angiogenic molecule *VEGF*. In contrast, non-magnetic scaffolds showed no significant effects in response to magneto-mechanical actuation over respective static controls suggesting that there are no direct effects of the magnetic field on cellular responses. The results corroborate that controlled shape changes achieved via application of an external magnetic field to a ferromagnetic fibrous scaffold can be used to induce changes in cellular behavior. This concept can be exploited in developing biomedical devices with the potential for controlled actuation in vivo.

## Figures and Tables

**Table 1:** In vitro cell mechanotransduction studies carried out during the past five years (2019–2014). Bone marrow mesenchymal stem cells, BMSCs; bone marrow-derived mesenchymal progenitor cells, BMPCs; h, human; r, rat; mouse, m; murine embryonic stem cells, mESC; human acute monocytic leukemia cell line, THP-1; human embryonic stem cell-derived mesenchymal progenitors, hES-MP.

Scaffold	Cell Type	Stimulation Device	Mechanical Stimulation	Effect of Mechanical Stimulation on Bone-Related Transcriptions, Growth Factors, and Proteins	Ref
PU foam	hES-MP	Perfusion bioreactor	Flow rate: 3.47 mL·min^−1^	*ALP* and DNA significantly increased.	[24]
PCL-TCP	hBMSC	Perfusion bioreactor	Cyclic compression: 0.22%, 1 Hz, 4 h per day. Biaxial rotation: 5 rpm, 90° angle, flow rate of 3.8 mL·min^−1^.	Combination of cyclic compression and biaxial rotation showed upregulation of *ALP* by 3.2–fold, *OPN* by 2.4–fold, *OCN* by 10–fold, *COL1A1*– by 2–fold, *Runx2* by 3.5–fold	[25]
PCL	MC3T3-E1	Loading device	Compression at 10 millistrain, 1 Hz, 0.5 h and 2 h for 4/8/12 days	*ATP* release was induced by overloading and further increased the intracellular calcium concentration	[26]
Chitosan-HA super-porous hydrogel	hBMSC & THP-1	Perfusion bioreactor	Bidirectional perfusion, flow rate of 6 mL·h^−1^, 28 days	*ALP*, *BSP*, *COL1A1*, *ON*, *TRAP*, and *IL-6* upregulated, *Runx2, OPN*, and *OCN* not affected	[27]
HA-PLG	hBMSC	Perfusion bioreactor and XYZ shaker	Continuous perfusion at 3 mL·min^−1^ or XYZ shaker, 30 rpm, max tilt angle of 4.5°	*SP7* and *COL1A1* upregulated after 7 days in the continuous perfusion. No differences at 14 and 21 days. Ca deposited increased in the continuous perfusion at 7, 14, and 21 days compared to the shaker.	[28]
PU-based meniscus	rBMSC	Perfusion bioreactor	Flow rate of 10 mL·min^−1^ and hydrodynamic pressure (2 s per cycle) at 10 mL·min^−1^, 0.5 Hz, at hydraulic pressure: 0–60 or 0–120 mmHg.	*ALP* upregulated by 4, 6.5, 6.2-fold and *OC* upregulated by 1, 5.5, 5.7-fold for 0–60 mmHg group after 1,2,3 weeks, respectively. No noticeable effect for 0–120 mmHg group.	[29]
Gelatin-coated porous polyurethane	hBMPC	Perfusion bioreactor	Single perfusion session for 2 h, flow rate of 2.5 mL·min^−1^ on day 5 or 7.	*ALP* and *Runx2* activity increased on day 7 compared to day 5 and static controls. *BMP-2* increased on day 5 compared to day 7 and static controls.	[30]
PU-based	hBMSC	Perfusion bioreactor	Perfusion at 10 mL·min^−1^ with 10% cyclic compression 0.5 or 5 Hz.	*Runx2* inhibited in 5 Hz group on day 7. *COL1A1* upregulated on day 7 and *OCN* enhanced on day 14 for 0.5 Hz.	[31]
Octacalcium phosphate and gelatin	mBMSC	Loading device	Cyclic compressive strains of 20, 40, and 60%, 0.75 Hz, 4 h for 3 or 7 days.	*ALP* activity decreased with increasing strains. *COL1* was not significantly affected. *OPN* and *OCN* upregulated for the 20% cyclic strain group compared to the control and other strain groups.	[32]
Collagen I	rBMSC	A piezo-type mechanical stimulator	Sinusoidal compressive deformation, 0.2% at 0.2, 2, 10, 20, 40, 60 Hz, 3 min per day.	Ca deposition (optically monitored) increased on days 5–7 and reached the highest value for 2 Hz (1.5-fold) on day 14.	[33]
Collagen I	mESC	Loading device (six-well loading plate)	5% cyclic compressive strain, 1 Hz, 2 loading cycles, 4 h followed by 16-h rest (total: 40 h).	*Runx*2 upregulated in loaded constructs on days 15, 20, and 30.	[34]
PLGA	hMSC	Bioreactor	Dynamic tensile stimulation: 1% strain at 1 Hz for 90 min twice daily for 28 days.	*COL1, COL2, fibronectin* and *Tenascin-C* were upregulated.	[35]
PCL	MC3T3-E1	Perfusion bioreactor	Computational fluid dynamic (CFD) analysis: flow rate of 1 mL·h^−1^, wall shear stress 3 Pa.	*ALP* per DNA values in perfusion culture increased from 14 to 28 days.	[36]
Partially deproteinized bone	Osteoblast-like rat osteosarcoma cells (ROS17/2.8)	3D fluid flow cell culture system	Shear stress of 0.8 Pa, loading for 1 h/2 h/4 h followed by statically incubated for 23 h/22 h/20 h.	*ALP*, *LRP5, β-catenin* and *Wnt3A*, upregulated in all loading conditions.	[37]
Collagen hydrogel	hMSCs	Magnetic Force Bioreactor	Magnetic field of 25 mT and 1 Hz for 1 h a day for 28 days	2.4-fold increase in mineralization and matrix density	[38]
Collagen I	mESCs	Loading device (six-well loading plate)	5% cyclic compressive strain, 1 Hz, 2 loading cycles, 4 h followed by 16-h rest (total: 40 h).	*Connexin-43* was 12-fold on day 5 and 5-fold on day 30. *Oct-4* increased on day 5. *Runx2* increased on day 7.	[39]

**Table 2:** Fiber volume fractions, cross-sectional shapes and mean fiber inclination angles (angle between the fiber axis and the through-thickness direction) for 444 and 316L networks [41,42].

Fiber Network	Fiber Volume Fraction (%)	Fiber Cross-Sectional Shape	Mean Fiber Inclination Angle to the Vertical (°)
444	15.7 ± 1.0	Rectangular (60 × 100 µm^2^)	81.87 ± 0.21
316L	15.4 ± 0.9	Hexagonal (side length 20 µm, diagonal length 40 µm)	83.49 ± 0.09

## Supplementary Materials

The following are available online at https://www.mdpi.com/2077-0383/8/10/1522/s1, Figure S1: Representative immunofluorescence images showing side views of the magnetically-actuated (M) and static (S) 444 fiber networks on day 21 of culture. FITC- phalloidin and DAPI were used to stain the actin cytoskeleton green and the nuclei blue, respectively. Figure S2: (**a**) Representative immunofluorescence images showing magnetically-actuated (M) and static (S) 316L fiber networks on day 21 of culture. FITC- phalloidin and DAPI were used to stain the actin cytoskeleton green and the nuclei blue, respectively; (**b**) Fluorescence imaging of mineralization obtained using the OsteoImage Mineralization Assay and calcium-rich deposits stained with Alizarin red, for actuated and non-actuated 316L networks on day 21 of culture. Figure S3: Schematic representation of a single fiber under an applied magnetic field *B*. Figure S4: Experimental setup for measuring the deflections of 444 fiber networks.
