# Supplementary material for: Stimulation of Human Osteoblast Differentiation in Magneto-Mechanically Actuated Ferromagnetic Fiber Networks
Source: J Clin Med. 2019 Sep 22;8(10):1522. doi: 10.3390/jcm8101522 (PMC6833056; doi:10.3390/jcm8101522)
Supplement: Supplementary file 1 [file jcm-08-01522-s001.pdf]

Stimulation of Human Osteoblast Differentiation in Magneto-mechanically Actuated Ferromagnetic Fibre Networks

Galit Katarivas Levy [1,*], Mark A. Birch [2], Roger A. Brooks [2], Suresh Neelakantan [1,3] and Athina E. Markaki [1,*]

[1] Department of Engineering, University of Cambridge, Trumpington Street, Cambridge CB2 1PZ, UK; gk406@cam.ac.uk (G.K.L.); sureshn@iitd.ac.in (S.N.); am253@cam.ac.uk (A.E.M.)
[2] Division of Trauma and Orthopaedic Surgery, Department of Surgery, University of Cambridge, Addenbrooke's Hospital, Hills Road, Cambridge, CB2 2QQ, UK; mab218@cam.ac.uk (M.A.B.); rb10003@cam.ac.uk (R.A.B.)
[3] Department of Materials Science and Engineering, Indian Institute of Technology Delhi, Hauz Khas, New Delhi 110 016, India.

1. Cell infiltation in 444 fibre networks

Samples were prepared according to the procedure described in section §2.3 *Cell adhesion and cytoskeleton organization*. Longitudinal sections of the scaffolds were obtained using shears metal tin snips cutter scissors. The fluorescence images were obtained using a Zeiss Axio-Abserver.Z1 fluorescence microscope. Figure S1 shows representative side-views of cell infiltration in actuated and non-actuated 444 networks after 21 days of culture. It can be seen that the inter-fibre spaces are filled with cells throughout the network thickness.

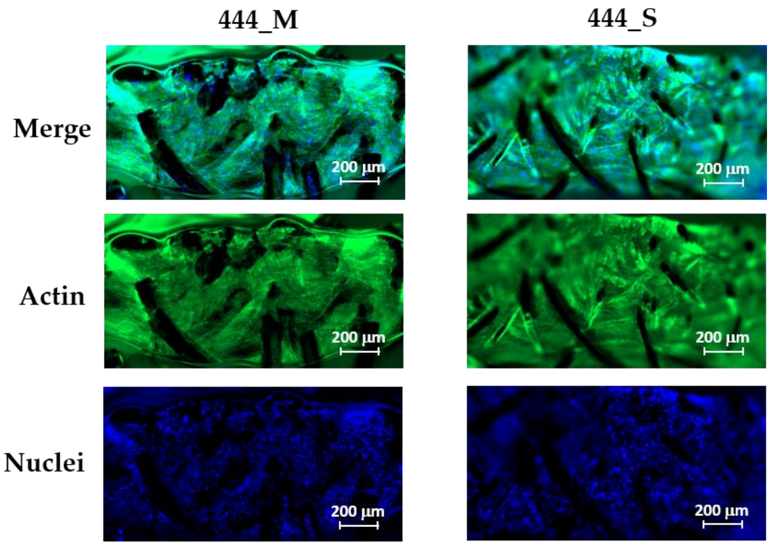

Figure S1. Representative immunofluorescence images showing side views of the magnetically-actuated (M) and static (S) 444 fibre networks at day 21 of culture. FITC- phalloidin and DAPI were used to stain the actin cytoskeleton green and the nuclei blue, respectively.

2. Cell adhesion, cytoskeleton organization and mineralization in 316L fibre networks

Samples were prepared according to the procedure described in §2.3 *Cell adhesion and cytoskeleton organization* and §2.4. *Cell Mineralization*. Figure S2(a) shows the actin filaments and cell nuclei for both static and actuated 316L networks. As in the case of the 444 fibre networks, at day 21 of culture, the cells have populated the inter-fibre spaces of the networks and show a well-organized actin cytoskeleton. Figure S2(b) shows mineralization at day 21 of culture for static and actuated 316L networks. The presence of bone mineral (green fluorescence staining) and a dense calcium-rich (red) layer are evident in both groups.

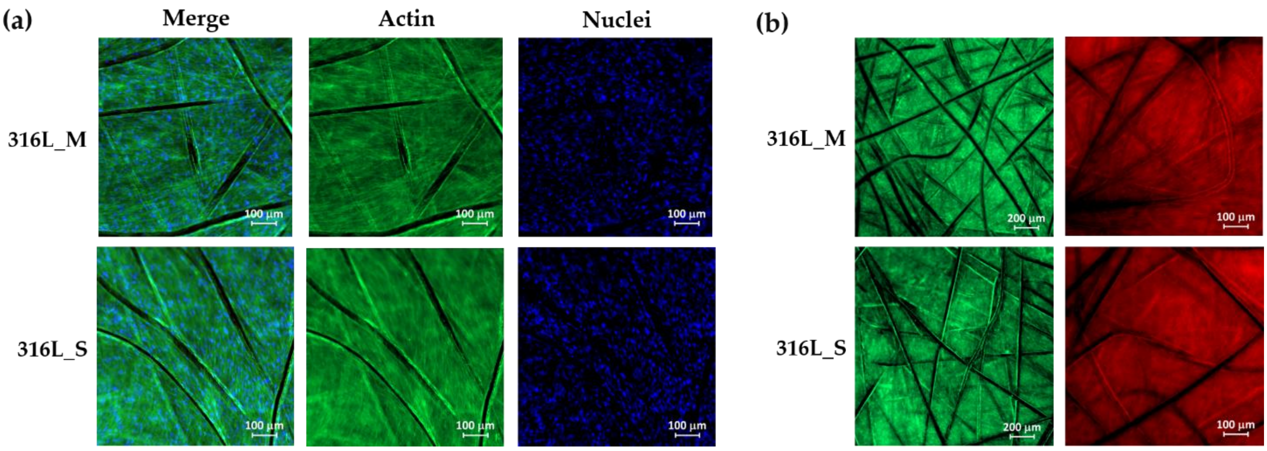

Figure S2. (**a**) Representative immunofluorescence images showing magnetically-actuated (M) and static (S) 316L fibre networks at day 21 of culture. FITC- phalloidin and DAPI were used to stain the actin cytoskeleton green and the nuclei blue, respectively; (**b**) Fluorescence imaging of mineralization obtained using the OsteoImage Mineralization Assay and calcium-rich deposits stained with Alizarin Red, for actuated and non-actuated 316L networks at day 21 of culture.

3. Magneto-mechanical Network Deflection

Consider a single ferromagnetic fibre inclined at an angle θ to a magnetic field *B* (Figure S3). Assuming that the fibre is fully magnetized along its length, the torque τ (bending moment) acting on the fibre is given by

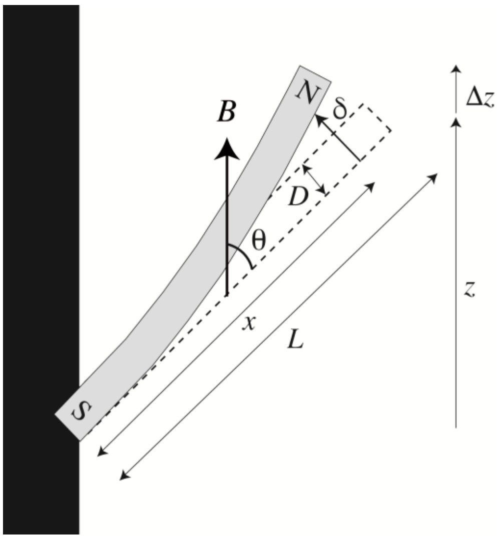

Figure S3. Schematic representation of a single fibre under an applied magnetic field *B*.

$$\vec{\tau} = \vec{m}_{\mathrm{tot}} \times \vec{B} = m_{\mathrm{tot}} B \sin\theta, \quad (1)$$

where $\vec{m}_{\mathrm{tot}}$ is the vector sum of all the magnetic moments within a cylindrical fibre and is related to the saturation magnetization $\vec{M}_s$ by

$$\vec{m}_{\mathrm{tot}} = \frac{\pi D^2}{4} L \vec{M}_s \quad (2)$$

in which D and L are the fibre diameter and length respectively. The deflection, δ, at a distance x (Figure S3) along an end-loaded cantilever beam subjected to the magnetically induced bending moment can be written as [10]

$$\delta = \frac{8M_s B\sin\theta}{3E_f D^2}(3Lx^2 - x^3) \qquad (3)$$

in which E_f is the fibre Young's modulus.

Representing each fibre segment as two beams, cantilevered at the vertices and loaded at the center point [10], the relative net extension of the networks ΔZ/Z in the direction of the applied field can be obtained by summing the contributions from the deflections of individual fibres as if they were free-standing, taking into account their orientation distribution $P(\theta)$:

$$\frac{\Delta Z}{Z} = \frac{\int_0^{\pi/2} \Delta z P(\theta)\mathrm{d}\theta}{\int_0^{\pi/2} zP(\theta)\mathrm{d}\theta} = = \frac{\int_0^{\pi/2} \delta\sin\theta\, P(\theta)d\theta}{\int_0^{\pi/2} \frac{L}{2}\cos\theta\, P(\theta)d\theta} = \frac{\int_0^{\pi/2} \frac{8M_s B\sin^2\theta}{3E_f D^2}\left(3\left(\frac{L}{2}\right)^3 - \left(\frac{L}{2}\right)^3\right)P(\theta)\,\mathrm{d}\theta}{\int_0^{\pi/2} \frac{L}{2}\cos\theta P(\theta)\,\mathrm{d}\theta}$$

$$\therefore \frac{\Delta Z}{Z} = \frac{4MsB}{3E_f}\left(\frac{L}{D}\right)^2 \frac{\int_0^{\pi/2} \sin^2\theta\ \ \mathrm{P}(\theta)\mathrm{d}\theta}{\int_0^{\pi/2} L\cos\theta\,\mathrm{P}(\theta)\,\mathrm{d}\theta} \qquad (4)$$

Equation (4) shows that networks with high fibre segment aspect ratios L/D are more readily deformable (i.e. can exhibit high relative length changes in the direction of the applied magnetic field ΔZ/Z).

Experimental Setup for Magneto-mechanical Network Deflection:

Rectangular beam network samples were magneto-mechanically actuated by being placed between the pole pieces of a 0.7 Tesla water-cooled DC electromagnet, with the field direction parallel to the axis of the specimen. The setup is shown in Figure S4.

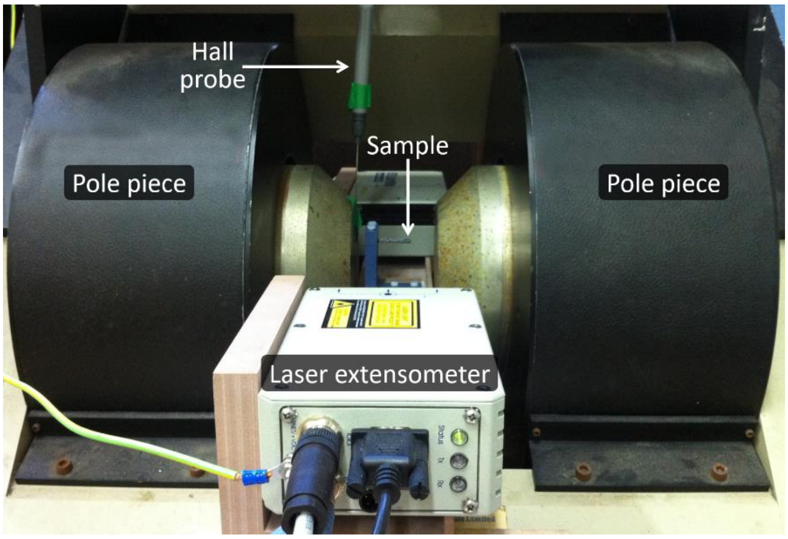

Figure S4. Experimental setup for measuring the deflections of 444 fibre networks.
